# Skin Cancer Classification With Deep Learning: A Systematic Review

**DOI:** 10.3389/fonc.2022.893972

**Published:** 2022-07-13

**Authors:** Yinhao Wu, Bin Chen, An Zeng, Dan Pan, Ruixuan Wang, Shen Zhao

**Affiliations:** ^1^ School of Intelligent Systems Engineering, Sun Yat-Sen University, Guangzhou, China; ^2^ Affiliated Hangzhou First People’s Hospital, Zhejiang University School of Medicine, Zhejiang, China; ^3^ School of Computer Science and Technology, Guangdong University of Technology, Guangzhou, China; ^4^ School of Electronics and Information, Guangdong Polytechnic Normal University, Guangzhou, China; ^5^ School of Computer Science and Engineering, Sun Yat-Sen University, Guangzhou, China

**Keywords:** generative adversarial networks, convolutional neural network, deep learning, skin cancer, image classification

## Abstract

Skin cancer is one of the most dangerous diseases in the world. Correctly classifying skin lesions at an early stage could aid clinical decision-making by providing an accurate disease diagnosis, potentially increasing the chances of cure before cancer spreads. However, achieving automatic skin cancer classification is difficult because the majority of skin disease images used for training are imbalanced and in short supply; meanwhile, the model’s cross-domain adaptability and robustness are also critical challenges. Recently, many deep learning-based methods have been widely used in skin cancer classification to solve the above issues and achieve satisfactory results. Nonetheless, reviews that include the abovementioned frontier problems in skin cancer classification are still scarce. Therefore, in this article, we provide a comprehensive overview of the latest deep learning-based algorithms for skin cancer classification. We begin with an overview of three types of dermatological images, followed by a list of publicly available datasets relating to skin cancers. After that, we review the successful applications of typical convolutional neural networks for skin cancer classification. As a highlight of this paper, we next summarize several frontier problems, including data imbalance, data limitation, domain adaptation, model robustness, and model efficiency, followed by corresponding solutions in the skin cancer classification task. Finally, by summarizing different deep learning-based methods to solve the frontier challenges in skin cancer classification, we can conclude that the general development direction of these approaches is structured, lightweight, and multimodal. Besides, for readers’ convenience, we have summarized our findings in figures and tables. Considering the growing popularity of deep learning, there are still many issues to overcome as well as chances to pursue in the future.

## 1 Introduction

Given the rising prevalence of skin cancer and the significance for early detection, it is crucial to develop an effective method to automatically classify skin cancer. As the largest organ of the human body (1), the skin shoulders the responsibility of protecting other human systems, which increases its vulnerability to disease (2). Melanoma was the most common cancer in both men and women with approximately 300,000 new cases (3) diagnosed globally in 2018. In addition to melanoma, two other major skin cancer diseases, basal cell carcinoma (BCC) and squamous cell carcinoma (SCC), also had a relatively high incidence, with over 1 million cases in 2018 (4). As (5) reported, more skin cancers are diagnosed each year than all other cancers combined in the United States. Fortunately, if detected early, the chances of cure will be greatly improved. According to (4), melanoma has a 5-year survival rate of 99% when it does not metastasize. If it metastasizes to other organs in the body, its survival rate reduces to 20%. However, because early indications of skin cancer are not always visible, diagnostic results are often dependent on the dermatologist’s expertise (6). For inexperienced practitioners, an automatic diagnosis system is an essential tool for more accurate diagnoses. Beyond that, diagnosing skin cancer with naked eyes is highly subjective and rarely generalizable (7). Therefore, it is necessary to develop an automatic classification method for skin cancer that is more accurate, less expensive, and quicker to diagnose (8). Besides, implementing such automated diagnostic systems can effectively minimize mortality from skin cancers, benefiting both patients and the healthcare systems (9).

However, owing to the complexity and diversity of skin disease images, achieving automatic classification of skin cancer is challenging. First of all, different skin lesions have lots of interclass similarities, which could result in misdiagnosis (10). For example, there exist various mimics of BCC in histopathological images, such as SCC and other skin diseases (11). As a result, it is difficult for the diagnosis systems to effectively discriminate skin malignancies from their known imitators. Secondly, several skin lesions differ within their same class in terms of color, feature, structure, size, and location (12). For example, the appearance of BCC and its subcategories is almost different. This makes it difficult to classify different subcategories of the same category. Furthermore, the classification algorithms are highly sensitive to the types of camera devices used to capture images. When the test images come from a different domain, their performance suffers (13).

Although traditional machine learning approaches are capable of performing well in particular skin cancer classification tasks, these algorithms are ineffective for complicated diagnostic demands in clinical practice. Traditional machine learning methods for skin cancer diagnosis typically involve extracting features from skin-disease images and then classifying the extracted features (14). For example, ABCD Rule (15), Menzies Method (16), and 7-Point Checklist (17) are effective methods for extracting various features from skin disease images. The handcrafted features are then classified using several classification methods such as SVM (18), XGBoost (19), and decision tree (20). Due to the restricted number of selected features, machine learning algorithms can often only classify a subset of skin cancer diseases and cannot generalize to a broader range of disease types (21). Besides, given the wide variety of skin cancers, it is not effective to identify each form of cancer solely based on handcrafted features (22).

Without the need for domain expertise and feature extraction, deep learning algorithms have been widely used for skin cancer classification in recent years; however, there are still several difficulties and challenges ahead. Compared with traditional machine learning methods, deep learning algorithms can analyze data from a large-scale dataset faster and more accurately, which allows them to effectively extract relevant characteristics (23). At the same time, deep learning algorithms can also aid clinicians in more thorough data analysis and examination of test results (24). A number of studies, such as (25–27) demonstrated that deep learning algorithms can diagnose at a level comparable to that of a dermatologist. However, these algorithms still have many obstacles to becoming a complete diagnostic system. Firstly, data imbalance and the lack of a large volume of labeled images have hindered the widespread use of deep learning methods in skin cancer classification (12). When these algorithms are used to classify skin cancers that are rare in the training dataset, they frequently result in a misdiagnosis (28). Furthermore, when working with high-resolution images (such as pathological images) with millions of pixels, the deep learning models often result in significant computing costs and additional training time (29). Besides, different noises will be generated as a result of the various conditions (such as different imaging devices, backgrounds). Therefore, the robustness and generalization ability of these algorithms should also be taken into account (30).

These years, a number of reviews that detail the diagnostic breakthroughs in skin cancer classification have been published; however, no review has provided a specific analysis of frontier challenges in skin cancer classification tasks, such as data imbalance and limitation, domain adaptability, model robustness, and model efficiency (31). reviewed the recent developments in skin lesion classification using dermoscopic images (32). presented a detailed overview of studies on using CNNs to classify skin lesions (33). showed how the use of CNNs in correctly identifying skin cancer has developed (34). presented a review of different machine learning algorithms in dermatology diagnosis, as well as some of the obstacles and limitations (12). and (28) summarized a number of deep learning-based approaches for skin cancer classification, as well as various challenges and difficulties (35). provided an in-depth review of the current articles about melanoma classification and compared their results with human experts (36). summarized the latest CNN-based methods in skin lesion classification by utilizing image data and patient data (37). provided a review of deep learning-based methods for early diagnosis of skin cancer. We present these relevant surveys with details and highlights in **Table 1**. By summarizing the previous reviews, we find that all of the preceding publications methodically studied a specific topic in skin cancer classification. However, most of them treated skin cancer classification as a classical classification problem, without addressing the model’s significant practical constraints in clinical work, such as data imbalance and limitation, cross-domain adaptability, model robustness, and model efficiency. Although several earlier reviews summarized some of the methods to solve the abovementioned frontier problems, their summaries were incomplete. Some novel techniques were not covered, such as pruning, knowledge distillation, and transformer. Therefore, in this review, we comprehensively summarize the frontier challenges in skin cancer classification and provide corresponding solutions by analyzing articles published until the year 2022. It gives readers in-depth information on the advances and limitations of deep learning in skin cancer classification and also provides different ideas for researchers to improve these algorithms.

**Table 1:** A summary of the current review related to skin cancer classification.

Ref.	Title	Venue	Remarks
(32)	Skin Cancer Classification Using Convolutional Neural Networks: Systematic Review	Journal of Medical Internet Research	This study presents a detailed overview of studies on using CNNs to classify skin lesions.
(31)	Techniques and algorithms for computer aided diagnosis of pigmented skin lesions—A review	Biomedical Signal Processing and Control	This paper gives a review of the recent developments in skin lesion classification using dermoscopic images.
(33)	Classification of Skin cancer using deep learning, Convolutional Neural Networks -Opportunities and vulnerabilities-A systematic Review	International Journal for Modern Trends in Science and Technology	This article reviews the development of deep learning for skin cancer classification tasks.
(34)	Machine Learning in Dermatology: Current Applications, Opportunities, and Limitations	Dermatology and Therapy volume	This paper reviews the fundamentals of machine learning and its wide range of applications in dermatology.
(12)	Artificial intelligence-based image classification methods for diagnosis of skin cancer: Challenges and opportunities	Computers in Biology and Medicine	This review discusses the developments in AI-based methods for skin cancer diagnosis, as well as challenges and future directions to enhance them.
(35)	Skin cancer classification *via* convolutional neural networks: systematic review of studies involving human experts	European Journal of Cancer	This paper analyses studies comparing AI–based skin cancer classifiers with dermatologists.
(37)	Skin Cancer Detection: A Review Using Deep Learning Techniques	International Journal of Environmental Research and Public Health	This paper provides a review of deep learning-based methods for early diagnosis of skin cancer.
(36)	Integrating Patient Data Into Skin Cancer Classification Using Convolutional Neural Networks: Systematic Review	Journal of Medical Internet Research	This review summarizes the latest CNN-based methods in skin lesion classification by utilizing image data and patient data.
(28)	Skin disease diagnosis with deep learning: A review	Neurocomputing	This paper analyses several deep learning algorithms for diagnosing skin diseases from a variety of perspectives based on the challenges at hand.

The rest of this paper is organized as follows: first of all, Section 2 introduces three types of dermatological images and several popular public datasets. In Section 3, we review several typical CNN frameworks and frontier problems with their corresponding solutions in skin cancer classification tasks. A brief conclusion is given in Section 4.

## 2 Dermatological Images and Datasets

High-quality images of skin diseases are important for both dermatologists and automated diagnostic systems. On the one hand, dermatologists rely on high-resolution (HR) images to make diagnoses when direct observation is impossible (38). This is especially common in telemedicine, medical consultations, and regular clinics (39). On the other hand, training reliable algorithms has always necessitated the use of high-quality data. In particular, deep learning algorithms always need a vast volume of labeled data for a better accuracy (28). As a result, high-quality dermatological images are critical for both clinical diagnosis and the design of new algorithms. In this section, we go over three different types of images commonly used in skin cancer diagnosis, as well as some public datasets.

### 2.1 Dermatological Images

**Figure 1:**
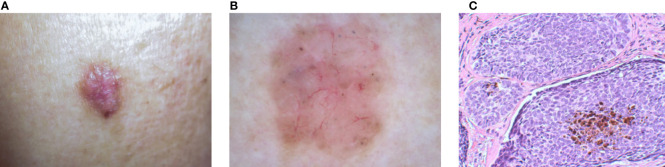
Examples of three types of dermatological images of BCC to show their differences and relationships: **(A)** Clinical image. **(B)** Dermoscopy image. **(C)** Histopathological image.^1^

The three main types of image modalities used to diagnose skin diseases are clinical images, dermoscopy images, and histopathological images (see **Figure 1**). Clinical images are frequently captured by mobile devices for remote diagnosis or as medical records. Dermoscopy images and histopathological images are commonly utilized in clinical diagnosis to assess the severity of the illness. In the next part, we introduce them separately.

#### 2.1.1 Clinical Images

Clinical images are obtained by photographing the skin disease site directly with a camera. They can be used as a medical record for patients and provide different insights for dermoscopy images (12). The biggest issue of utilizing clinical images for skin cancer classification is that they include limited morphological information while also introducing considerable inaccuracies into the diagnostic results, owing to the effect of diverse imaging settings (such as lighting, angle, and so on) (40).

#### 2.1.2 Dermoscopy Images

Dermoscopy images are captured with dermoscopy, a type of optical observation tool used to assess the fine details of skin diseases (41). Clinicians frequently utilize dermoscopy to diagnose benign nevi and malignant melanoma (42). It serves as a bridge between clinical and pathological aspects, and thus dermoscopy is often referred to as a dermatologist’s stethoscope. Dermoscopy images provide a clear visualization of the skin’s surface and are used to analyze the color and microstructure of the epidermis (43). For some skin diseases, there are already numerous diagnostic guidelines based on dermoscopy images (44), for example, the ABCD Rule Law (15), the CASH Rule Law (45), and the Menzies Method (16). When using dermoscopy images for skin cancer diagnosis, the range of structures that can be observed is limited, and its diagnostic accuracy is occasionally affected by the experience of dermatologists (46).

#### 2.1.3 Histopathological Images

Histopathological images were obtained using microscopes to scan tissue slides and then digitalize as images (28). They are utilized to show the vertical structure and complete internal characteristics of the diseased tissue. In the clinic, pathological examinations serve as the “gold standard” for diagnosing almost all types of cancers, as they are often used to distinguish between types of cancers and guide appropriate treatment plans based on pathological changes. However, different histopathological images of skin cancer exhibit different morphologies, scales, textures, and color distributions, which makes it difficult to find a common pattern for diagnosis (12).

### 2.2 Datasets

To create a trustworthy and robust skin cancer classification system, a variety of datasets with all kinds of dermatological images are required. As the need for medical imaging resources in academia grows, more and more datasets are becoming publicly available. To provide readers with a reference, we introduce several commonly used skin-disease datasets in the next part, along with the works based on these datasets.

#### 2.2.1 PH^2^ Dataset

The PH^2^ dataset is constructed by (47) to support the research of classification and segmentation methods. It contains 200 color dermoscopy images (768 × 560) of three types of skin diseases, including common nevi, atypical nevi, and melanomas. Besides, it contains complete medical annotations, such as lesion segmentation results and pathological diagnosis.

PH^2^ is frequently used as a dataset for testing the diagnostic algorithms of skin disease. For example (48), used the SegNet framework to automatically diagnose and segment the dermoscopic images in *PH^2^* and finally obtained the classification accuracy of 94% (49). proposed a novel deep convolutional network for feature extraction and classification of skin lesions. The model was mainly divided into three stages. The first stage was for data augmentation and image contrast enhancement. The second stage used CNN to extract information from the boundary of the lesion area. The third stage used Hamming distance to fuse and select features obtained with pretrained Inception v3. Finally, the model obtained a classification accuracy of 98.4%, 95.1%, and 94.8% on the PH^2^, ISIC-2016, and ISIC-2017 datasets, respectively (50). proposed a Multi-Focus Segmentation Network for skin cancer disease segmentation tasks based on the PH^2^ dataset by utilizing feature maps of different scales. Two boundary attention modules and two reverse attention modules were utilized to generate skin lesion masks. Finally, the experimental results revealed that the proposed method achieved a dice similarity coefficient of 0.954 and an IoU index of 0.914 on the PH^2^ dataset. In addition to the above works, the PH^2^ dataset is being utilized by an increasing number of algorithms to validate their effectiveness and accuracy.

#### 2.2.2 MED-NODE Dataset

The MED-NODE Dataset^3^ is collected by the Department of Dermatology of the University Medical Center Groningen (UMCG), which contains 170 digital images of melanoma (51) and nevi case (52). It is used to build and evaluate the MED-NODE system for detecting skin cancer with macroscopic images (53).

On the MED-NODE dataset, a variety of approaches provided significant classification results. For example, in order to improve the generalization ability of the model and alleviate the problem of data imbalance (54), proposed a model for melanoma classification based on transfer learning and ensemble learning. Finally, the model achieved 93% classification accuracy on the MED-NODE dataset, surpassing other state-of-the-art methods (55). applied AlexNet for the skin cancer classification task by using three different transfer learning methods, including fine-tuning the weight parameters of the model, replacing the classification layer function, and performing data augmentation on the original dataset. In the end, they achieved an accuracy of 96.86% on the MED-NODE dataset. Then (56), used two additional networks for the skin cancer classification task, including ResNet-101 and GoogleNet. Finally, experiment results revealed that GoogleNet achieved the best classification accuracy of 99.29% on the MED-NODE dataset. It can be seen that various convolutional neural networks have obtained decent classification results on this dataset; however, the number of skin disease images included is relatively restricted.

#### 2.2.3 HAM10000 Dataset

The HAM10000^4^ dataset was collected by the International Skin Imaging Collaboration (ISIC) to solve the problem of data imbalance and data limitation in skin-disease datasets. It contains 10,015 dermoscopic images with seven representative diseases in pigmented skin lesions: nematode disease and intraepithelial carcinoma, basal cell carcinoma, benign keratoid lesions, cutaneous fibroma, melanoma, melanocyte nevi, and vascular lesions (including hemangiomas, purulent granulomas, and subcutaneous hemorrhage) (57, 58).

The HAM10000 dataset is widely used by many scholars due to its diversity of skin lesions. For example (25), used four novel deep CNN models, DenseNet-201, ResNet-152, Inception-v3, and InceptionResNet-v2 to classify eight different types of skin cancers on the HAM10000 and PH^2^ datasets. Finally, experimental results indicated that the diagnostic level of these CNN models exceeds the dermatologists in terms of ROC AUC score (59). trained 30 different models on the HAM10000 dataset to explore the classification performance of different models. At the same time, they also used two locally interpretable methods GradCAM and Kernel SHAP techniques to observe the mechanism of the classification model. Finally, the model achieved an average AUC of 0.85 (60). designed a method for classifying seven skin diseases that used ensemble learning and the one-versus-all (OVA) strategy. Finally, they achieved a classification accuracy of 0.9209 on the HAM10000 dataset (61). obtained the best classification result by combining Inception ResNet-v2 with Soft-Attention mechanism on the HAM10000 dataset, with an accuracy of 0.934, an AUC of 0.984, and an average precision of 0.937. With the in-depth study of skin cancer classification tasks by scholars, more and more novel classification methods are being tested on the HAM10000 dataset for a better comparison, where the adoption of the Soft-Attention module yields the best classification results.

#### 2.2.4 Derm7pt Dataset

The Derm7pt dataset contains approximately 2,000 clinical and dermoscopy color images of skin disease, as well as structured information for training and assessing CAD systems. It serves as a database for analyzing the prediction results of the seven-point malignancy checklist of skin lesion (62).

Due to the multimodal information contained in the Derm7pt dataset, it has gradually been widely used to test various multitask networks. When releasing the dataset (62), also proposed a multitask network for predicting melanoma with seven-point checklist criteria and diagnostic results. The model used different loss functions to handle different input modalities, while being able to make predictions on missing data at the output. Finally, the model achieved a classification accuracy of 73.7% on the Derm7pt dataset, also benchmarking the approach. To increase its interpretability (63), created a multitask model based on the Derm7pt dataset to illustrate the mechanism between different tasks. Learnable gates were used in the model to show how the method used or combined features from various tasks. This strategy may be used to investigate how CNN models behave, potentially enhancing their clinical utility (64). proposed a deep convolutional network for skin lesion classification on the Derm7pt dataset. Meanwhile, they implemented regularized DropOut and DropBlock to increase the model’s generalization capabilities and reduce overfitting. In addition, to address the dataset’s imbalance and limitation, they devised a novel loss function that assigns different weights to various samples, as well as an end-to-end cumulative learning technique. Finally, the method achieved excellent classification performance on the Derm7pt dataset and ISIC dataset while with low computational resources. The release of the Derm7pt dataset has a great boost in promoting the use of multimodal data in skin cancer classification tasks, as well as new ideas and solutions.

#### 2.2.5 BCN20000 Dataset

The BCN20000^5^ dataset comprises 5,583 skin lesions and 19,424 dermoscopic images taken using high-resolution dermoscopy. They were all gathered between 2010 and 2016. At the same time, the collector employed a variety of computer vision techniques to remove noise, background, and other interference from the images. Finally, they were carefully reviewed by numerous experts to ensure the diagnosis’ validity (65).

BCN20000 is commonly utilized in skin cancer classification and segmentation tasks as part of the dataset for the ISIC-2019 competition. For example, in order to protect the data privacy and avoid data abuse (66), proposed a Distributed Analytics method for distributed training of skin disease images, which ensures that the training data remains in the original institution. Finally, after training on the BCN20000 dataset, the model achieves classification accuracy comparable to the centralized distribution. To evaluate the robustness of different CNN models (67), generated a series of out-of-distribution (OOD) images by using different data augmentation methods based on BCN20000, HAM10000, and other skin-disease datasets. This method establishes a benchmark for OOD testing and considerably facilitates the clinical use of skin cancer classification methods. Specially, by using different data augmentation methods with an ensemble learning strategy (including EfficientNets, SENet, and ResNeXt101_wsl) (68), achieved the first-place classification result with a balanced accuracy of 74.2% on the BCN20000 dataset.

#### 2.2.6 ISIC Dataset

To reduce skin cancer mortality while promoting the development and use of digital skin imaging (69), the International Skin Imaging Collaboration (ISIC) has established a publicly available skin disease dataset^6^ for the computer science community around the world. Currently, ISIC Archive comprises over 13,000 representative dermoscopic images from clinical facilities throughout the world, all of which have been inspected and annotated by experts to ensure image quality (70).

The majority of studies that utilized the ISIC dataset focused on skin cancer classification and segmentation tasks, with the binary classification task being the most popular. For example (71), designed different modules based on VGGNet for skin disease classification (melanoma or benign) and benchmarked for the ISIC-2016 dataset. In the end, results showed that this method obtained excellent performance with an accuracy of 0.8133 and a sensitivity of 0.7866 (51). achieved the best classification results with an AUC of 0.911 and balanced multiclass accuracy of 0.831 on three skin cancer classification tasks of ISIC-2017 by using an ensemble of ResNet-50 networks on normalized images (72). used ensemble learning with a stacking scheme and obtained the classification results with an accuracy of 0.885 and an AUC of 0.983 in the ISIC-2018 competition (73). employed two bias removal techniques, “Learning Not to Learn” (LNTL) and “Turning a Blind Eye” (TABE), to alleviate irregularities in model predictions and spurious changes in melanoma images. Among them, the LNTL method combined a new regularization loss with a gradient inversion layer to enable the model to debias the CNN’s features in backpropagation. The TABE method reduced biases by using different auxiliary classifiers to identify biases in features. Finally, the experimental results revealed that TABE had a more effective denoising effect, with an improvement of 11.6% in the AUC score benchmark on the ISIC dataset. Since the ISIC dataset is widely used in competitions and research, readers can find more methods for comparison on the competition leaderboard or on the Internet.

**Table 2** summarizes the above datasets to show the different characteristics between them. What we summarized are the most common datasets in the skin cancer classification task and may not be the most exhaustive summary. Readers can find more datasets from various sources online. At the same time, it can be seen from the above summary that most of the images in the skin-disease dataset are dermoscopic images, while clinical images and histopathological images are still relatively rare. Furthermore, most skin-disease datasets have a relatively small number of images compared with datasets of natural images, which poses certain challenges for skin cancer classification tasks.

**Table 2:** Characteristics of different skin-disease datasets.

Dataset	No. of images	Modality of images	No. of lesion types	Image format	Published year	Goal of publication
PH^2^	200	Dermoscopic	3	.bmp	2013	To facilitate the development of computer-aided diagnosis systems in the segmentation and classification of melanoma.
MED-NODE	170	Macroscopic	2	.jpg	2015	To build and evaluate the MED-NODE system for detecting skin cancer with dermoscopic images.
HAM10000	10,015	Dermoscopic	8	.jpg	2018	To address the small size and insufficient diversity of images in the skin-disease dataset.
Derm7pt	2,000	Dermoscopic Structured data	15	.jpg	2018	As a database for the analysis of a seven-point malignant checklist for skin lesions.
BCN20000	19,424	Dermoscopic	9	.jpg	2019	Used to analyze skin cancer lesions in hard-to-diagnose locations such as nails and mucous membranes.
ISIC Archive	>13,000	Dermoscopic	9	.jpg, DICOM	2016–2020	To reduce skin cancer mortality while promoting the development and use of digital skin imaging.

## 3 Methods for Typical and Frontier Problems in Skin Cancer Classification

In the past few years, many scholars have been working on developing computer-aided diagnostic (CAD) systems for skin cancer classification. Before the emergence of deep learning, the CAD systems were primarily designed by machine learning (ML) algorithms (74). However, due to the complexity of feature engineering and limitations of handcrafted features, these ML-based methods can only diagnose a subset of skin diseases. Deep learning algorithms, on the other hand, can automatically learn semantic features from large-scale datasets with higher accuracy and efficiency. As a result, deep learning-based methods such as Convolutional Neural Network (CNN) have been used to solve the great majority of skin cancer classification problems in recent years and obtained satisfactory results.

However, as we dig deeper into the challenges of skin cancer classification, it appears that they are not as straightforward as the challenges in the non-medical domain (e.g., ImageNet, PASCAL-VOC, MS-COCO) (75) (12). Firstly, many datasets of skin images are imbalanced due to the disproportions among different skin cancer classes, which increases the risk of misdiagnosis by the diagnostic system. Also, since correct annotation needs a great amount of expertise knowledge and is time-consuming and labor-intensive, many datasets only provide a limited number of images (e.g., the ISIC dataset is the largest publicly available skin disease dataset until now, which contains about 13,000 skin images). As a result, more labeled data is required to design a more accurate system. Besides, when the amount of training data is insufficient, the model’s generalization performance degrades. In addition, different noises generated by different devices or different shooting conditions also bring biases to the model, resulting in a reduction in diagnosis. Furthermore, the operational efficiency and resource consumption of the model also limit its clinical implementation on various medical devices.

As a result, in the following part, we present a complete overview of the use of deep learning methods in skin cancer classification. We begin by introducing the use of typical CNN frameworks in skin cancer classification, then review the frontier challenges in skin cancer classification and provide related solutions. We summarize these methods in **Tables 3**–**6**. Among them, **Table 3** summarizes the use of typical frameworks in skin cancer classification, as well as their highlights and limitations. **Tables 4**–**6** summarize the approaches to address the frontier issues of data imbalance and limitation, model generalization ability and robustness, and model computational efficiency in skin cancer classification. At the same time, we list publications based on the same or similar dataset together to make it easier for readers to compare different approaches.

**Table 3:** References of skin cancer classification with typical CNN frameworks.

Ref.	Dataset	CNN Architecture	Highlights	Limitations	Performance
(76)	Self-collected dataset	Deep Belief Network, SVM	By combining deep belief networks and SVM classifiers to handle skin cancer diagnosis tasks with limited datasets, as well as outliers and erroneous data.	The generalization ability of the model is limited.	Accuracy: 0.89
(77)	Self-collected dataset	Resnet-34, ResNet-50 ResNet-101 and ResNet-152	Proposed how to improve deep learning-based dermoscopy classification and dataset creation.	Data from more modalities, such as the patient’s medical history, information on other symptoms, are not considered.	Accuracy: 0.85
(78)	Online repositories and the Stanford University Medical Center	Inception-v3	Used a CNN framework to train a large-scale skin disease dataset and achieve superior results on par with dermatologists. The method was also developed for mobile devices.	More research is required to assess its performance in clinical practice. At the same time, this method is limited to some extent by the amount of data.	Accuracy: 0.6375 (avg.)
(79)	MED-NODE	Deep CNN	Compared with previous methods, it directly used CNN to automatically extract features for skin disease images, also had a higher classification accuracy.	Due to the large noise interference of clinical images, there are still some misclassifications.	Accuracy: 0.81 PPV: 0.75, NPV: 0.86
(71)	ISIC-2016	VGG-16	It reduces the training time of the model by using the transfer learning strategy while obtaining higher sensitivity and precision.	It is prone to overfitting due to the limited amount of training images.	Accuracy: 0.813 Sensitivity: 0.787
(80)	ISIC-2017, IAD	Inception-v2	Introducing sonification into the diagnosis of skin cancer lesions to improve the sensitivity of the model.	Differences in the diagnosis of pathologists can affect the prediction results of the model.	AUC: 0.976 Sensitivity: 0.86 Specificity: 0.91
(27)	ISIC-2017	DenseNet, Dual Path Nets Inception-v4, Inception-ResNet-v2MobileNetV2, PNASNet, ResNet SENet, Xception	By analyzing 13 factors from 9 different models, they systematically evaluated the factors influencing the choice of CNN structure.	The dataset used in this article is too limited, and it only focuses on the melanoma classification task.	Top accuracy: 0.827
(81)	IAD	VGG-19	Adopted VGG-19 network to evaluate the thickness of melanoma for the first time.	There are no more pre-training methods utilized for comparison, and precisely predicting melanoma thickness would be more clinically significant.	Accuracy: 0.872 Specificity: 0.840
(82)	Derm7pt	Inception-v3	A multi-task network was designed to classify the seven-point checklist and skin disease diagnosis. Different loss functions were also designed to handle different input modalities, such as clinical and dermoscopic images, and patient diagnostic results.	Some criteria of the 7-point checklist are unable to be distinguished.	Accuracy: 0.737
(60)	HAM10000	Deep CNN models	Proposed a method combining CNN with one-versus-all (OVA) for skin disease classification.	The model has not been tested on datasets from various domains and may have a large variance.	Accuracy: 0.929
(83)	HAM10000 ISIC-2019	ResNeXt, SeResNeXt, DenseNet Xception, and ResNet	Adopted a grid search strategy to find the best ensemble learning methods for skin cancer classification.	The amount of training data is still insufficient, and most of models employed in ensemble learning are from the same network architecture.	Accuracy: 0.88 F1 score: 0.89

**Table 4:** Different methods for solving data imbalance and data limitation.

Ref.	Dataset	Highlights	Limitations	Performance
(84)	ISIC-2017	By coupling seven GANs to generate seven skin-disease images. At the same time, they improved the efficiency of the model by making the initial layers of GANs share the same parameters.	The model was unable to distinguish the lesion area well when it was mixed with the skin surface, and artifacts such as human hair can also affect the generation of new images.	Accuracy: 0.816 AUC: 0.88
(85)	ISIC-2018	Proposed a GAN architecture that was customized to the style of skin lesions. At the same time, it can generate higher resolution and more diverse skin disease images by adjusting the progressive growth structure of the generator and discriminator in the GAN network.	The content of the GAN-generated synthetic dataset was not complicated enough when compared with the original dataset, and it was also not diverse enough.	Accuracy: 0.952
				Sensitivity: 0.832
				Specificity: 0.743
(86)	ISIC-2018	Utilized conditional generative adversarial networks (CGAN) to extract key information from all layers to generate skin lesion images with different textures and shapes while ensuring the stability of training.	The amount of data used for training was relatively limited.	Accuracy: 0.941
				Precision: 0.915
				Recall: 0.799
(87)	ISIC Archive	Explored four types of data augmentation methods and a multiple layers augmentation method in melanoma classification.	The data augmentation methods evaluated in this paper were limited and not validated on a large amount of datasets.	Accuracy: 0.829
(88)	HAM10000	They adopted a variational autoencoder network to get domain-dependent noise vectors. Also, a student-like distribution was employed to increase image diversity, and an auxiliary classifier was used to create images of certain classes.	Due to the specificity of medical images, different image generation models may generate skin disease images that did not belong to the same class.	Accuracy: 0.925
(89)	HAM10000	It combined the attention mechanism with PGGAN to obtain global features of skin lesions images, also introduced the Two-Timescale Update Rule to generate features with high fine-grainedness, while increasing the stability of GAN.	Due to the limitation of hardware conditions, this data augmentation method was only evaluated on the resolution of 256 × 256, rather than the original resolution of 600 × 450 in HAM10000 dataset.	AUC: 0.793
(90)	HAM10000	Proposed a class-weighted loss function and a focal loss to overcome the problem of data imbalance.	There is no artifact removal for the images in the training dataset, which leads the model to be biased. Also, it has a relatively high computational complexity.	Accuracy: 0.93
				Recall: 0.86
(91)	HAM10000	A novel loss function was combined with the balanced mini-batch logic of the data level to alleviate the imbalance problem of the dermatology dataset.	The classification accuracy for rare skin diseases with limited data needs to be improved further.	Accuracy: 0.8997 Recall: 0.8613
	ISIC-2019			
(92)	HAM10000	Proposed a two-stage technique for determining the appropriate augmentation procedure for mobile devices.	Given the particularity of lightweight CNN, more data augmentation methods and data need to be considered to alleviate the problem of overfitting.	Accuracy: 0.853
(93)	PAD-UFES	Designed two algorithms based on evolutionary algorithm and also applied weighted loss function and oversampling to alleviate the problem of data imbalance.	A larger dataset was necessary to improve the performance further.	Accuracy: 0.92
				Recall: 0.94
(94)	PH^2^	Proposed novel a data augmentation method based on a oversampling technique (SMOTE).	The proposed data augmentation method was not validated in the deep learning architectures, and experiments on larger datasets were also required.	Accuracy: 0.922
				Sensitivity: 0.808
				Specificity: 0.951

**Table 5:** Different methods for improving model generalization ability and robustness.

Ref.	Dataset	Highlights	Limitations	Performance
(95)	DermIS DermQuest	Investigated the advantages of large-scale supervised pre-training for medical imaging applications.	In addition to the analysis of the weights and features of the model, it is necessary to conduct a comprehensive analysis of other features such as network structure to explore the importance of pre-training.	Accuracy: 0.871 (DermIS)
				Accuracy: 0.974 (DermQuest)
(96)	HAM10000 MoleMap	Proposed transfer learning and adversarial learning in skin disease classification to improve the generalization ability of models to new samples and reduce cross-domain shift.	When the data domain and target domain are significantly different, the method’s overall accuracy suffers.	Accuracy: 0.909
				AUC: 0.967
(97)	HAM10000	Performed adversarial training on MobileNet and VGG-16 using the innovative attacking models FGSM and PGD for skin cancer classification.	The number of datasets tested for this experiment is very limited, and there may be local optimizations.	Accuracy: 0.7614
(98)	ISIC-2016	Proposed a comprehensive deep learning framework combining adversarial training and transfer learning for melanoma classification. At the same time, focal loss was introduced to iteratively optimize the network to better learn hard samples.	This method does not consider more types of skin diseases, and it had a high computational cost.	Accuracy: 0.812
				Sensitivity: 0.918
(99)	ISIC2017 HAM10000	Presented a Multi-view Filtered Transfer Learning approach to extract useful information from the original samples for domain adaption, thereby improving representation ability for skin disease image.	The effectiveness of this domain adaptation method should be validated on more dermatology datasets.	Accuracy: 0.918
				AUC: 0.879
(100)	ISBI-2017, PH^2^	Proposed an adversarial training method combined with attention module to enhance the robustness of the model in skin-disease classification and segmentation.	Due to the limited amount of training data and the unclear boundaries of skin disease images, the model still suffers from under-segmentation and over-segmentation.	Accuracy: 0.968
				Sensitivity: 0.962
				Specificity: 0.941
(101)	ISIC-2018	Using seven universal adversarial perturbations to investigate the vulnerability of the classification model.	This method does not perform adversarial training on more skin disease datasets, so the robustness of its model needs to be further improved.	Accuracy: 0.873
(102)	ISIC-2019	Proposed Monte Carlo dropout, Ensemble MC dropout, and Deep Ensemble for uncertainty quantification.	Further optimization of the robustness of the model is required, and the model should also be tested for noise detection to provide a confidence score.	Accuracy: 0.90
				AUC: 0.945
(103)	ISIC Archive MED-NODE Dermofit	Proposed a transfer learning method to address the shortage of data in skin lesion images. Also, they utilized a hybrid deep CNN model to accurately extract features and ensure training stability while avoiding overfitting.	The model requires a considerable amount of computational resources while also lacking the diversity of domains.	Accuracy: 0.853
				F1 score: 0.891
(104)	HAM10000, Dermofit, Derm7pt, MSK PH^2^, SONIC, UDA	Proposed to improve the generalization performance of the model by combining data augmentation and domain alignment. Designed a Bayesian generative model for continual learning based on a fixed pretrained feature extractor.	Due to the privacy of medical images, this trained model may underperform on ethnic groups with a small proportion of the population.	Accuracy: 0.670
(105)	Skin7, Skin40		To increase the method’s overall performance, better pre-training of the extractor can be investigated.	Mean class recall: 0.65

**Table 6:** Different methods for improving model efficiency.

Ref.	Dataset	Highlights	Limitations	Performance
(106)	Self-collected	Proposed a knowledge distillation method to transfer knowledge between various models simultaneously.	The proposed method sacrifices local accuracy for higher global accuracy, with some additional classification errors on local objects.	Accuracy: 0.75
(107)	Public repositories	Proposed a MobileNet-based classification method and successfully deployed it on an Android application.	To improve the model’s classification accuracy, more sophisticated sampling strategies and data preprocessing can be adopted.	Accuracy: 0.944
(108)	HAM10000	Presented an assessment of the effectiveness for the attention module and self-attention module in skin cancer classification based on ResNet architecture.	Only limited number of attention mechanisms are used for comparison.	Accuracy: 0.622 (attention)
				Accuracy: 0.737 (self-attention)
(109)	HAM10000	Proposed a weight pruning strategy for lightweight neural networks to make up for the accuracy loss and improve model performance and reliability in the skin cancer classification.	The proposed pruning method is only validated on the skin disease dataset, and more kinds of medical images are needed to validate its effectiveness.	Accuracy: 0.975
	SH-11			AUC: 0.931
(110)	HAM10000, PH^2^, Dermofit	Designed a new pruning method “MergePrune” to reduce the computational cost of retraining the network by combining pruning and training into a single stage.	To assess this strategy, more domain data is needed, such as clinical images, patient meta-data.	Accuracy: 0.776 (avg.)
	Derm7pt, MSK, UDA			
(111)	ISIC-2017	Proposed a classification method that incorporated the attention residual learning (ARL) mechanism to EfficientNet for skin cancer diagnoses.	The interpretability of the model needs to be further strengthened.	Accuracy: 0.873
				AUC: 0.867
(112)	ISIC-2017	Three different lightweight networks MobileNet, MobileNetV2, and NASNetMobile were were evaluated for skin cancer classification.	The number of lightweight networks and hyperparameters used for testing are relatively restricted.	Accuracy: 0.82
				Precision: 0.812
(113)	ISIC-2017, PH^2^	Proposed an MT-TransUNet network to segment and classify skin lesions simultaneously.	The model finds it difficult with low-contrast skin disease images, and its segmentation performance is vulnerable to occlusions in the skin image.	Accuracy: 0.912
(114)	PH^2^, DermQuest	Built a pruning framework to simplify the complicated architectures by choosing the most informative color channels in skin lesion detection. Also, it carried out a hardware-level analysis of the complexity of different skin cancer classification networks.	The proposed method works well for simple networks, but it may not perform as well for more complicated networks.	Accuracy: 0.9811 (*PH^2^*)
				Accuracy: 0.9892 (DermQuest)
(115)	SD-198, SD-260	Proposed a knowledge distillation method based on curriculum training to distinguish herpes zoster from other skin diseases.	It requires manual tuning of hyperparameters according to different models and datasets.	Accuracy: 0.935
(116)	DermIS, DermQuest, DermNZ, “11K Hands”	Proposed an expert system “i-Rash” based on SqueezeNet to classify four skin diseases.	More clinical data and skin-disease images are needed to further improve the generalization of the model.	Accuracy: 0.972
				Sensitivity: 0.944
				Specificity: 0.981

### 3.1 Typical CNN Frameworks for Skin Cancer Classification

During the early stages of the development of CNN, people usually used self-building networks for a specific task. For example (76), presented a self-supervised model for melanoma detection. Firstly, a deep belief network and self-advised SVM were used to train the labeled and unlabeled images. After that, a bootstrap approach was used to randomly choose the training images for the network to improve the generalization ability and decrease the redundancy of the model. Experiments showed that the proposed method outperformed other methods like KNN and SVM. Then (79), designed a simple CNN network work for detecting melanoma. Firstly, all input images were preprocessed to eliminate the effects of noise and artifacts. The processed images were then fed into a pretrained CNN to detect if they were melanoma or benign. Finally, experiment results showed that CNN outperformed other classification methods.

With the development of deep learning, various well-known networks, such as VGGNet (117), GoogleNet (118), and ResNet (119), have been applied to skin cancer classification with favorable results. The most landmark work was (78). It was the first time that a CNN has been utilized to train large-scale clinical images for skin cancer classification. They designed an end-to-end network for automated skin cancer classification using Inception v3. A total of 129,450 clinical images with 2,032 distinct skin diseases were utilized for training the model. Meanwhile, to make use of the fine-grained information in taxonomy structure, they proposed a disease partitioning algorithm to divide skin cancers into fine-grained classes (e.g., melanoma was subdivided into amelanotic melanoma and acrolentiginous melanoma). In the end, the results of the experiments indicated that the skin cancer classification system could attain diagnostic levels equivalent to dermatologists. In the same year (71), successfully implemented VGGNet for skin lesion classification (melanoma or benign) and benchmarked for ISIC datasets by using dermoscopic images. In this study, they designed three different modules based on VGG-16 as comparison. The first module trained the network from initial weights. The second module used pretrained VGG-16 for training and then used the current dataset to train the fully connected classifier. The third module also used transfer learning to train the network, but weights in the high-level part of the convolutional layers were initialized from the first module. In the end, results showed that the third module obtained excellent performance in skin cancer classification. Different from previous classification tasks (81), utilized a CNN framework (VGG-19) for the first time to evaluate the thickness of melanoma. They began by locating the lesion and cropping the region of interest (ROI). To solve the problem of data limitation and data imbalance; they then employed the Synthetic Minority Over-sampling technique to generate synthetic samples. After that, the pretrained VGG-19 was used for the thickness prediction. Finally, the results demonstrated that the algorithm can estimate the thickness of melanoma with an accuracy of 87.5%. For the first time, a multitask network was proposed by (82) based on Inception v3 by utilizing three different modalities of data to predict seven-point criteria. In addition, they designed a multimodal–multitask loss function to tackle the combinations of input modalities, which was also able to make predictions with incomplete information. Finally, results showed the superior performance in classifying skin lesions and the seven-point criteria. Besides, the proposed method had the ability to identify discriminating information and generate feature vectors for image retrieval (80). built two systems for skin disease classification based on the novel deep learning algorithms. Additionally, they added a sonification-derived layer to increase the sensitivity of the model. In the first system, a CNN architecture was proposed based on Inception v2 to identify skin diseases (benign or malignant) with dermoscopic images. The second system transformed the feature representation generated in the preceding system into sound data. Then, this sound information was then put into a machine learning classifier or translated to spectrograms for further analysis. In the end, both systems performed exceptionally well in terms of classification and sonification. After the deep learning methods achieved excellent results in the skin cancer classification task (77), proposed how to improve deep learning-based dermoscopy classification and dataset creation. They analyzed four ResNet architectures in dermoscopic classification, namely, ResNet-34, ResNet-50, ResNet-101, and ResNet-152, to apprehend the mechanisms and certain error causes. First, four ResNet networks were trained at their best fits to see if the structural differences between the models would result in different classification results. After testing with several epochs, they found that the accuracy of different models tended to be consistent and varied with different hyperparameter settings. Meanwhile, they had a high level of stability during training. Therefore, the training errors of the classification models were attributed to incorrect annotations and the complexity of medical images.

Gradually, people discovered that applying a single CNN to a CAD system typically did not produce the desired results due to the large variances in deep neural networks. After that, ensemble learning was proposed as a way to limit the error generated by a single model by training multiple models and then combining their results to get the final classification results (27). compared the performance between ensemble models and a single model by utilizing nine different CNN architectures in skin cancer classification. After different comparative experiments, they found the significance of ensemble learning for obtaining optimal classification models. In addition, they investigated the effectiveness between two different selection strategies in ensemble learning: random selection and utilizing a validation set. For the smaller ensemble models, they found that the second method had more advantages, but the first was also effective. For the larger ensemble models, it was possible to get away with merely picking models arbitrarily. Based on the same method (60), proposed two different methods for skin cancer classification while reducing the complexity of the model by using an OVA strategy: i) alone CNN model and ii) the incorporation of seven CNN models. In the first method, images from the dataset were directly put into the single CNN model for the final prediction. In the second method, a one-versus-all (OVA) strategy was used to combine seven separate models with two classes to obtain the final prediction. Each class in this method was classified according to true and false labels, thus increasing the efficiency of the model. The results revealed that the second method outperformed the first in terms of classification accuracy (83). adopted a grid search strategy to find the best ensemble learning methods for the classification of seven skin lesions. During the training, five CNN networks, ResNeXt, SeResNeXt, ResNet, Xception, and DenseNet, were used as baseline. After that, two ensemble learning strategies, namely, average ensemble and weighted ensemble, were conducted to find the optimal model. In the end, results showed that the weighted ensemble model had more advantages than the average ensemble model.

### 3.2 Data Imbalance and Data Limitation in Skin Disease Datasets

Data imbalance and data limitation in skin disease datasets are common problems in the skin cancer classification tasks. In fact, benign lesions account for the majority of data in many skin disease datasets. Meanwhile, many skin disease datasets have large inequities in the number of samples among different skin disease classes. Only the common skin diseases, such as BCC, SCC, and melanoma, are included in the majority of skin disease datasets. Other skin cancer diseases (such as appendiceal carcinomas and cutaneous lymphoma) are relatively rare in these datasets, making it difficult for algorithms to classify them correctly (28). Besides, the skin lesions in most of the current datasets are from fair-skinned people, with only a few from dark-skinned people (12). It has been demonstrated that deep learning frameworks that have been validated for skin cancer diagnosis in fair-skinned people are more likely to misdiagnose those with different races or ethnicity (120). At the same time, the quantity of skin disease images is also relatively restricted. For example, ISIC-2020 (121) is the dataset with the largest number images so far, with about 30,000 skin disease images. Although large amounts of skin disease images can be obtained from websites or medical institutions without any diagnosis information, labeling them takes professional knowledge and can be extremely challenging and time-consuming. What is more, sufficient labeled data are a requirement for training a reliable model. When only a limited number of images are provided, overfitting is more likely to occur. As a result, for the skin cancer classification task, a considerable amount of labeled data is required.

Generative adversarial networks (GAN) are widely thought to be a preferable alternative, as they can generate artificial data to compensate for data imbalance in terms of positive and negative proportions, rare cases, and different people (84). designed a data augmentation method based on generative adversarial networks to address the shortcomings of skin lesion images in melanoma detection. Firstly, they utilized several data processing methods to locate and eliminate hairs and other artifacts of the input images. Then they used two convolutional GANs, namely, DCGANs, to generate 350 images of melanoma and 750 images of seborrheic keratosis. Finally, the results demonstrated that combining the processing module and generative adversarial networks resulted in superior performance when compared with other baselines. Although GAN is extensively employed for data augmentation, the images it generated are typically low-resolution. To overcome this issue (85), proposed a style-based GAN to generate more high-quality images in skin lesion classification. Then these synthetic images were added to the training set to the pretrained ResNet-50 model. The experiment showed that the proposed style-based GAN method outperformed other GAN-based methods in terms of Inception Score (IS), Fréchet Inception Distance (FID), precision, and recall. What is more, the accuracy, sensitivity, specificity, and other indicators of the classification model also improved. In (88), the author proposed a GAN-based framework “TED-GAN” to generate skin lesion images artificially in skin cancer classification. Instead of using random Gaussian distribution to sample the noise vector in GAN, they used informative noise that was obtained from a separate network for the first time to generate the medical images. TED-GAN had four parts: one variational auto-encoder, two GANs, and one auxiliary classifier. Firstly, an auto-encoder network was trained to get the vector containing the image manifold’s information. Then one of the GANs sampled output of the auto-encoder to ensure the stability of training and make it more convenient to use the domain information. After that, the other GAN obtained more training data from the prior GAN. In addition, an auxiliary classifier was added to this GAN network, then the two were trained together to generate images of various skin diseases. In the end, experiment results showed that TED-GAN had a positive effect on skin cancer classification as it provided more images for training. Although data augmentation methods such as GAN may successfully increase the number of skin cancer images and alleviate the problem of data imbalance, the generated data usually have identical distributions, limiting the improvement in model performance. To solve this issue (89), proposed a data augmentation method based on PGGAN, namely, SPGGAN, to generate skin lesion images with different types and data distributions. Firstly, an attention module was added into SPGGANs to obtain the global and local information from skin lesion images, also enabling PGGAN to generate more diverse high-quality samples. Then, the Two-Timescale Update Rule (TTUR) was added to SPGGANs to reduce the signal magnitude increase and hence enhance the stability of the model. Finally, experiments showed that the GAN-based data augmentation approach can lead to an improvement in the classification in terms of accuracy, sensitivity, F1 score, and other metrics. Since skin lesions often contain irregular boundaries, varied textures, and shapes, it makes the training of the GAN framework sometimes unstable. To address this issue (86), utilized conditional generative adversarial networks (CGANs) to extract key information from all layers and generate skin lesion images. The proposed CGAN has two modules: a generator module and a discriminator module. The generator module was to extract useful features from high-level and low-level layers and generate synthetic images. The discriminator module was to accurately map latent feature components by combining auxiliary information with training images. After that, augmented images with original datasets were put into the pretrained ResNet-18 network for the classification task. Experiments showed that this model achieved superior results compared with other datasets.

Another popular method for resolving data imbalance is to apply weights to various samples in the loss function. The goal is to calculate the losses differently depending on whether the samples are in the majority or minority. For example (90), proposed an end-to-end framework for classifying seven skin lesions in the HAM10000 dataset. Especially, a class-weighted learning strategy was utilized to overcome the problem of data imbalance in the dataset by assigning different weights to different lesion classes in computing the loss function. Meanwhile, focus loss was used to further increase the model’s classification performance. It concentrated training on tough examples, preventing the classifier from being overwhelmed by easy samples. Experiment results revealed that the model obtained an average accuracy of 93%, outperforming dermatologists’ 84% accuracy. Although the problem of data imbalance can be alleviated through the design of the loss function, there exists a problem of slow learning of the minority classes. To solve the issue (91), proposed a hybrid strategy for skin cancer classification. It combined a loss function method at the algorithm level with a balanced mini-batch logic method for real-time image augmentation at the data level. By applying the balanced mini-batch and real-time image augmentation method, the new loss function can improve its learning ability in minority samples, thereby improving training efficiency. When compared with the previous strategy, this method improved the learning effectiveness of minority classes on an imbalanced dataset by increasing m-Recall by 4.65% and decreasing the standard deviation of recalls by 4.24%. In addition to designing a new loss function (93), also designed two new algorithms based on evolutionary algorithms, the Mixup Extrapolation Balancing (MUPEB) and the Differential Evolution (DE), to solve the problem of data imbalance in melanoma classification. The MUPEB method included a set of operations to mix and interpolate the dataset until it was balanced. The DE method mixed and combined three random images with varied clinical information to achieve data balance. Apart from that, weighted loss function and oversampling were also used to alleviate data imbalance. In the end, this algorithm increased the model’s classification precision and recall by 1% and 11%, respectively.

Data augmentation is an ideal solution to artificially increase the amount of data by generating new data points from existing data. It scales the number of images by random rotating, padding, rescaling, flipping, translation, etc. At the same, with the development of technology, various novel approaches for data augmentation have been presented in skin cancer classification (58, 122). released the HAM10000 dataset by natural data augmentation; the images of skin lesions were captured at various magnifications or angles, or with multiple cameras. To evaluate the effectiveness of data augmentation methods while determining the most effective method (87), explored four types of data augmentation methods (geometric transformation, adding noise, color transformation, and image mix) and a multiple-layer augmentation method (augmented images by more than one operation) in melanoma classification. The first step was to preprocess the images to remove artifacts such as body hair on the images. Then each augmentation method was assessed to decide the optimal augmentation method. In the end, they found that single-layer augmentation outperformed multiple-layer augmentation methods. Besides, the region of interest (ROI)-mix method achieved the best performance compared with other approaches (92). proposed a two-stage strategy data augmentation method on mobile devices successfully with limited computing resources. The first stage was to search the optimal augmentation method in the Low-Cost-Augment (LCA) space. The second stage was to fine-tune the deep CNNs with augmented images and choose the model with the highest accuracy. Finally, the augmented images were trained with EfficientNets, which resulted in better accuracy and computational efficiency. Different from previous data augmentation methods (94), proposed a novel Synthetic Minority Oversampling Technique (SMOTE) to solve the problem of image scarcity and imbalance in the skin lesion dataset. Firstly, all images in the PH^2^ dataset were preprocessed for ensuring cleaning. Then in the data augmentation stage, the covariance matrix (CM) was exploited by SMOTE to find dependent connections between attributes. Then they built surrogate instances based on the estimated CM to balance the number of minority class and majority class. Finally, all augmented images were utilized to train the SqueezeNet and it resulted in a significant improvement in terms of accuracy, sensitivity, specificity, and F1 score.

### 3.3 Poor Generalization Ability Across Different Domains

In the skin cancer classification task, the generalization ability of the model is often inferior to that of an experienced dermatologist. Firstly, owing to the small scale of skin image datasets, even if a large amount of similar data is artificially generated, the overfitting problem still exists. Secondly, the majority of research exclusively focuses on dermatological images taken using standardized medical equipment, such as dermoscopic and histological images (78). Little research has been conducted on dermatological images captured by other devices. When a trained model is applied to a new dataset with a different domain, its performance suffers significantly.

Transfer learning (TL) is commonly utilized for improving the generalization ability of computer-aided diagnostic systems in test data. The fundamental idea of TL is to preserve information gained while addressing a problem and implement it to a new but relevant problem (52). It can not only drastically reduce the time overhead and labor cost associated with partial repetitive labor but also compensate for the flaw in the skin disease datasets (96). presented two methods to improve the generalization ability of models to new samples and reduce cross-domain shift. The first method used a transfer learning strategy with two steps to acquire new knowledge from diverse domains. It began with pretraining on ImageNet and fine-tuned the model with a single skin dataset. In the end, they used the target set to fine-tune the model to get the prior information. The second method used a pixel-wise image synthesizing adaptation method to transfer the features between the source domain and target domain. In comparison to the previous transfer learning approach, this method was semi-supervised and did not need any labels for domain adaptation. Finally, cross-domain experiments showed that in order to improve classification performance, the proposed methods had the ability to transform images between different modalities. In order to solve the problem of class imbalance in skin lesion datasets, To address the problem of poor generalization performance due to low interclass variance and class imbalance in skin disease images (98), proposed a two-stage framework with adversarial training and transfer learning in melanoma detection. The first stage was to solve the data scarcity and class imbalance problem by generating underrepresented class samples. The second stage was to train deep neural networks for melanoma classification, by using newly synthesized images and original datasets. A focal loss was proposed to assist the model in learning from hard examples. In the end, results showed the significant improvement of the classification performance and superiority of the proposed method. With the application of transfer learning in skin cancer diagnosis, it has been discovered that most existing transfer learning methods only extract knowledge from the source data to learn, but many inaccurate samples that are very different from the target data are incorporated into the process. Meanwhile, most skin cancer classification methods simply learn from raw skin disease images, which makes information from different aspects (such as texture, shape, etc.) interfered by noise during the learning process. Therefore (99), proposed a multi-view-filtered transfer learning (MFTL) method to solve the poor scalability problem of skin cancer classification models. MFTL consisted primarily of two modules: a multi-view-weighing representation module and a filtered domain adaption module. The first module put the view weights obtained from the feature representation procedure to the final prediction. The second module selected key source information to transfer the knowledge between the source domain and target domain. Finally, the result showed a significantly improved performance in classifying melanoma and seborrheic keratosis. In (103), the author proposed a transfer learning approach to address the issue of insufficient data in the medical image datasets, as well as to improve the performance of other related medical image classification tasks. The proposed approach first trained deep learning models on a great amount of unlabeled images for a specific task, as the volume of unlabeled medical images has increased significantly. Then the models were fine-tuned on a relatively small-labeled dataset to perform the same task. Besides, they utilized a hybrid deep CNN model to accurately extract features and ensure training stability while avoiding overfitting. Experiments showed the effectiveness in the skin cancer and breast cancer classification in terms of classification accuracy, recall, precision, and F1 score. With the growing use of transfer learning in the field of computer vision, an increasing number of studies have proved that large-scale pretraining on natural images can be beneficial in a variety of tasks. However, research on medical images is still limited. With this purpose (95), investigated the advantages of large-scale supervised pretraining with three medical images: chest radiography, mammography, and dermatological images. Five tasks including in-domain performance, generalization under distribution shift, data efficiency, subgroup fairness, and uncertainty estimation were conducted to test if large-scale pretraining aided in the modeling of medical images. Finally, experiment results indicated that, despite significant differences from the pretraining data, employing larger pretraining datasets can achieve significant improvements across a wide range of medical disciplines. Besides, they discovered that pretraining at scale may allow downstream tasks to more effectively reuse deeper features.

In addition to TL, many novel methods such as adding innovative regularization terms, estimating model uncertainty, and lifelong learning models are beginning to be introduced into the skin cancer classification task to improve the generalization ability of the model across different domains (104). proposed a method that can improve the generalization ability of a model under limited samples by combining data augmentation and domain alignment. They observed in medical images that domain changes were compact and related to a certain extent. To be able to model such dependencies, the author introduced a dependency regularization term to learn a representative feature space that captured sharable information across different medical image domains. At the same time, a variational encoder was used to ensure that the latent features followed a predetermined distribution. Finally, through theoretical derivation, the author obtained the upper bound of empirical risk for any relevant target domain under this method, which alleviated the problem of overfitting. Finally, the generalization ability of the model was well confirmed on seven skin-disease datasets. In order to obtain the uncertainty quantification (UQ) of the deep learning model to prevent overfitting (102), proposed three indicators Monte Carlo (MC) dropout, Ensemble MC (EMC) dropout, and Deep Ensemble (DE) to solve this problem. They next presented a novel hybrid Bayesian deep learning model based on the three-way decision (TWD) theory to obtain the residual uncertainty after using the three methods of MC, EMC, and DE. It also enabled different UQ methods to be used in different neural networks or different classification stages. Finally, the experimental findings demonstrated that the proposed model can be employed efficiently in analyzing different stages of medical images, and the model’s uncertainty was accurately quantified. Since the deep learning model might forget much of the previous information while learning new data, updating the system with more new data would reduce the performance of the previous learning, which poses a greater challenge to the medical autonomous diagnosis system. To this end (105), designed a Bayesian generative model for continual learning based on a fixed pretrained feature extractor. Different from the previous continual learning method, which stored a small number of images for each old class, the proposed method stored the statistical information of each class based on the previous feature extractor, which can make the model naturally keep the knowledge of each old class from being used. Therefore, there was no need to store or regenerate old images. Finally, the model performed well on both the Skin7 and Skin40 datasets, and it was able to retain some images from previous classes during continual learning. The model’s scalability and generalization have been greatly enhanced.

### 3.4 Noises From Heterogeneous Devices and Images

Various noises obtained from heterogeneous sources and skin disease images pose challenges to the robustness of models in the task of skin cancer classification. When trained on high-quality skin lesion datasets, the deep learning model can reach the same diagnostic level as dermatologists, even surpassing them. However, since the skin cancer classification model is sensitive to images captured with different devices, lighting settings, and backgrounds, it frequently fails to obtain satisfactory classification results when tested with different images. Furthermore, photographic images (such as smartphone images) vary greatly in terms of zoom, perspective, and lighting, making classification much more difficult.

Therefore, many scholars have worked to integrate adversarial training into the field of skin cancer classification to enhance the robustness of the classification models. In (100), the author introduced a novel Attention-based DenseUnet (Att-DenseUnet) network combined with adversarial training for skin lesion segmentation and classification. With the addition of the attention module, the model can pay more attention to discriminative features while also successfully suppressing irrelevant features in the DenseBlocks output. In this way, the interference of artifacts on skin disease images is reduced. Att-DenseUnet had two main modules: Segmentor module and Discriminator module. The segmentor module was a U-Net shape structure, which contained a down-sampling path, up-sampling path, and related attention module to ensure the information transfer between different layers. Additionally, it adopted an attention module to focus on the essential features and speed up the training process. The discriminator module employed the adversarial training to impose the segmentor module to obtain diverse features with different sizes and shapes and direct the attention module to concentrate on the multiscale lesions. Besides, they used the adversarial loss to prevent overfitting by providing the regularization term for the networks. Finally, the results showed that this network achieved excellent performance and was robust enough for different skin image datasets. In clinical applications, it has been discovered that noises that are difficult for humans to detect frequently cause significant interference to the diagnostic model, limiting the utility of deep learning in the actual world. To improve the model’s robustness (97), performed adversarial training on MobileNet and VGG-16 using the innovative attacking models FGSM and PGD for skin cancer classification. Firstly, two white-box attacks based on Projected Gradient Descent (PGD) and Fast Gradient Sign Method (FGSM) were used to test the robustness of these models. Then, to increase the robustness of these models, the author did the adversarial training based on PGD against white-box attacks. In the end, the results showed that the robustness of these models significantly improved. To further increase the difficulty of adversarial attacks instead of simple adversarial attacks (101), used the more realistic and riskier Universal Adversarial Perturbation (UAP) to adversarially train seven classification models (VGG-16, VGG-19, ResNet-50, Inception ResNet-V2, DenseNet-21, and DenseNet-169). During the adversarial attack phase, the author used an iterative algorithm to generate perturbations for non-targeted and targeted attacks and the Fast Gradient Sign Method (FGSM) was used to generate perturbations for input images. After that, they conducted adversarial retraining to improve the robustness of these seven models. The results showed that these models were easily deceived when applied to adversarial attacks. In addition, they found the limited effect of adversarial retraining on non-targeted perturbations. Although adversarial retraining considerably lowered the vulnerability to adversarial perturbations in targeted attacks, it did not totally avoid it.

### 3.5 Toward Faster and More Efficient Classification Models

Although an increasing number of deep learning algorithms have been successfully applied to skin cancer classification with excellent classification results, the computational complexity of the model still needs to be considered. Firstly, due to improvements in imaging technology, many skin disease images with high resolution have large pixels. For example, histological scans are made up of millions of pixels, and their resolution is often larger than 50,000 × 50,000 (123). As a result, training them takes longer time and additional computing resources. Secondly, the computational complexity in the deep learning model is increasing as its accuracy improves, which demands their implementation to various medical equipment or mobile devices at a higher cost. Here we introduce three latest methods when designing an effective network for skin cancer classification.

Over the past few years, many Lightweight Convolutional Neural Networks have been designed and successfully applied in skin cancer classification to meet the demands of practical applications. Subsequently, many scholars used lightweight CNN for the task of skin cancer classification and successfully employed it to various mobile devices. For example (107), proposed an automated classification method based on MobileNet and successfully deployed it on an Android application or a website for public use. With the vigorous development of mobile health (mHealth), more and more mobile applications are designed for cancer classification and prediction. However, the application of automatic classification of skin cancer is still limited. To solve this problem (116), proposed an innovative expert system based on SqueezeNet, namely, “i-Rash,” to classify four classes of skin diseases in real time. Due to the limited size of “i-Rash” (i.e., 3 MB), identifying an unknown image for the system only took 0.09 s. Inspired by predecessors (111), proposed a novel method that incorporated attention residual learning (ARL) mechanism to EfficientNet with fewer parameters. Besides, they also investigated how the mechanism related to the existing attention mechanisms: Squeeze and Excitation (SE). Through the comparison of experimental results between models with and without SE, they speculated that the attention module accounts for a large portion of EfficientNet’s outstanding performance. What is more, the addition of ARL increased the accuracy of the EfficientNet and its variance. In (112), three different lightweight models (including MobileNet, MobileNetV2, NASNetMobile) were adopted for skin cancer classification. To find the model with the best performance, they tested a total of nine models with three different batch sizes. In the end, they found that the NASNetMobile model showed the best performance with a batch size of 16. Meanwhile, they benchmarked the lightweight models with fewer parameters and less computational time.

Pruning is an effective way to remove parameters from an existing network to maintain the accuracy of the network while increasing its efficiency (124). To enable CNN to be used in medical devices with limited power and resources (114), built a pruning framework to simplify the complicated architectures by choosing the most informative color channels in skin lesion detection. The proposed method is to achieve two purposes: removing redundant color channels and simplifying the whole network. Firstly, all color channels were put into the network. Then the weights that associated with the non-essential color channels were deleted to select the most informative color channel. After that, to generate a simplified network, they utilized CNN models as the target network and trained them on the chosen color channels. Besides, the requirements of these models were calculated from hardware perspectives to analyze the complexity of various networks. Finally, results showed that this color channel pruning strategy improved segmentation accuracy while also simplifying the network. Designing an efficient and generalizable deployment strategy is an extremely challenging problem for lightweight networks. To this end (109), proposed a weight pruning strategy for lightweight neural networks to make up for the accuracy loss and improve model performance and reliability in the skin cancer classification. Five lightweight CNNs, namely, SqueezeNet, MnasNet, MobileNetV2, ShuffleNetV2, and Xception, were investigated in this task. Firstly, a dense–sparse–dense (DSD) training strategy was used to avoid the underfitting and high bias of the networks. Then, a detailed analysis was used for building a pruning method including not just pruning connections with various relations but also reviewing a novel pruning mechanism that can remove the weights according to the distribution in each layer adaptively. In the end, the pruning strategy achieved higher accuracy and less computation compared with unpruned networks (110). designed a new pruning method “MergePrune” to reduce the computational cost of retraining the network by combining pruning and training into a single stage. Firstly, different units were assigned to learn each domain independently as they contribute differently to the classification result. Then, for one domain, determined culprit network units with high “culpability” scores were pruned and then reset and assigned to learn new domains. At the same time, non-culprit units were preserved. MergePrune was implemented to reduce the amount of computation and improve the efficiency of the classification model. Finally, the results showed that the network can perform accurately and effectively on real-world clinical imaging data with various domains, even with high pruning ratios.

Knowledge distillation is the process of distilling information from a huge model or group of models to a smaller model that could be successfully implemented with real-world restrictions (106, 125). proposed a knowledge distillation-based method that enabled to transfer knowledge between models simultaneously in skin cancer classification and brain tumor detection. Firstly, a pretrained ResNet-50 was chosen as a base model as its excellent performance out of the box. Then, with the significant degree of resemblance across the images in the medical image dataset, they let the knowledge transfer only between the two bottom-most layers. As a result, high-level visual comprehension was preserved, and information was added to the granular distinction in this way. The findings of the experiments were revealed in order to gather remote knowledge and enhance global accuracy; some local accuracy was lost. To improve the robustness and reduce the computational cost of the model (115), proposed a knowledge distillation method based on curriculum training in distinguishing herpes zoster from other skin diseases. Firstly, three kinds of model, namely, basic models, mobile models, and ensemble models, were chosen for benchmark. Then, to improve the performance of a single network, an ensemble knowledge distillation was utilized. This allowed the student network to learn more robust and representative features from the network while keeping a low computational cost. After that, they proposed curriculum training for ensemble knowledge distillation in order to distill ensemble teachers more efficiently with an adaptive learning technique. In the end, the results showed that the proposed method achieved improved performance while obtaining higher efficiency.

Transformer (126) is a deep learning model designed by the Google team in 2017 that was originally utilized in Natural Language Processing (NLP) and is now frequently employed in medical image processing, such as skin lesion images. It uses the self-attention mechanism to weigh the relevance of different parts of the input data series, resulting in shorter training periods and improved accuracy (126, 127). The introduction of the attention mechanism has generated great interest in the research community, but there is still a lack of systematic ways to select hyperparameters that guarantee model improvement. To this end (108), presented an assessment of the effectiveness for the attention module and self-attention module in skin cancer classifications based on ResNet architecture. Among the two modules, the attention module was used to recompute the features of the input tensor in each layer. The self-attention module was used to connect multiple positions of input images to obtain different representations of the input. In the experiment stage, the author investigated and compared a variety of alternative attention mechanisms with images from the HAM10000 dataset. In the end, the results showed that many of the self-attention structures outperformed the ResNet-based architectures, while containing fewer parameters. At the same time, applying the attention mechanism reduced the image noise; however, it did not behave consistently across different structural parameters. In solving the skin cancer classification problem, people often treat it as a simple classification task, ignoring the potential benefits of lesion segmentation. To this end (113), proposed an approach that combined the attention module with the CNN module for skin cancer classification. The CNN module was in charge of getting lesion texture information, while the attention module was responsible for obtaining context information such as the shape and size of the lesion. In addition, dual-task and attended region consistency losses were adopted to mediate the classification and segmentation heads without pixel-level annotation, which increased the robustness of the model when it trained with various augmented images. Finally, MT-TransUNet achieved excellent performance in the skin lesion segmentation and classification. At the same time, it preserved compelling computational efficiency and speed.

## 4 Conclusion

With the development of science and technology, the diagnosis accuracy and efficiency for skin cancer classification are constantly improving. In the previous clinical diagnosis scenarios of skin cancer, the final diagnosis often depends on the imaging quality and the experience of dermatological experts, which is highly subjective and has a high rate of misdiagnosis. With the advent of machine learning, various CAD systems have been designed to aid the dermatologists to diagnose skin cancer diseases. In some skin cancer classification tasks, these CAD systems achieved excellent performance by utilizing handcrafted features. Recently, with the success of deep learning in medical image analysis, several researchers have applied deep learning methods for skin cancer classification in an end-to-end manner and achieved satisfactory results. It is expected that in the future, artificial intelligence and the diagnosis of skin cancer diseases would become closely associated.

In this study, we present a comprehensive overview of the most recent breakthroughs in deep learning algorithms for skin cancer classification. Firstly, we introduced three different types of dermatological images used in diagnosis and some commonly used datasets. Next, we present the applications of typical CNN-based methods in skin cancer classification. After that, we introduce several frontier problems in the skin cancer classification task, such as data imbalance and limitation, cross-domain adaptability, model robustness, and model efficiency, along with relevant deep learning-based approaches. Finally, we provide a summary of the entire review. We draw the key information as follows:

- Skin cancer develops as a result of uncontrolled cell proliferation in the skin. It frequently appears on sun-exposed skin. The three major types of skin cancers are basal cell carcinoma (BCC), squamous cell carcinoma (SCC), and melanoma. Early skin cancer classification increases the chances of a successful treatment (refer to Section 1 for more information).
- Clinical images, dermoscopic images, and histopathological images are three common types of images used for skin disease diagnosis. Among them, the most common forms of images are dermoscopy images. With the growing need for medical imaging resources in academia, more and more datasets are becoming publicly available. We list several popular datasets for skin-disease images along with works based on these datasets. However, compared with natural image datasets, the diversity and quantity of skin-disease datasets are still very limited, which also brings great challenges to the automatic diagnosis of skin cancer (refer to Section 2 for more information).
- When using CNN-based methods for skin cancer classification, VGGNet, GoogleNet, ResNet, and their variants are the most often used deep learning models. Also, ensemble learning was proposed to limit the error generated by only a single model and achieved satisfactory results. Although various deep learning models have performed admirably on skin cancer classification tasks, several challenges still exist and need to be resolved, such as imbalanced datasets, a lack of labeled data, cross-domain generalization ability, noisy data from heterogeneous devices and images, and how to design effective models for complicated classification tasks. To address the challenges, methods include generative adversarial networks, data augmentation, designing new loss functions, transfer learning, continual learning, adversarial training, lightweight CNN, pruning strategy, knowledge distillation, and transformer. It can be expected that AI has the potential to play an active role in a paradigm shift in skin cancer diagnosis in the near future (refer to Section 3 for more information).

In comparison to other comparable reviews, this paper presents a comprehensive review in the topic of skin cancer classification with a focus on contemporary deep learning applications. It can be seen that the general evolutionary trend of these frameworks is structured, lightweight, and multimodal. With the help of this essay, one can gain an intuitive understanding of the core principles and issues in this field. Furthermore, anyone eager to engage in this field in the future should explore a number of different approaches to dealing with these issues. It is believed that the problems described above will become the research hotspots of scholars for a long time to come.

## Author Contributions

YW was responsible for writing the paper and supplementing materials. BC and RW were responsible for proposing amendments. AZ was responsible for revising and reviewing the article. DP was responsible for making the article more complete. SZ was the overall guide and was responsible for the whole project. All authors contributed to the article and approved the submitted version.

## Funding

This work was supported by the National Natural Science Foundation of China (Youth Project) under Grant 62101607 and National Natural Science Foundation of China under Grant 62071502.

## Conflict of Interest

The authors declare that the research was conducted in the absence of any commercial or financial relationships that could be construed as a potential conflict of interest.

## Publisher’s Note

All claims expressed in this article are solely those of the authors and do not necessarily represent those of their affiliated organizations, or those of the publisher, the editors and the reviewers. Any product that may be evaluated in this article, or claim that may be made by its manufacturer, is not guaranteed or endorsed by the publisher.

## Footnotes

1
https://dermnetnz.org/topics/basal-cell-carcinoma.

2
https://www.fc.up.pt/addi/ph2%20database.html.

3
https://www.cs.rug.nl/~imaging/databases/melanoma_naevi/.

4
https://dataverse.harvard.edu/dataset.xhtml?persistentId=doi:10.7910/DVN/DBW86T.

5
https://www.isic-archive.com/, 2019.

6
https://www.isic-archive.com/.
